# Cyclodextrin Enhances Corneal Tolerability and Reduces Ocular Toxicity Caused by Diclofenac

**DOI:** 10.1155/2018/5260976

**Published:** 2018-02-13

**Authors:** Hamdy Abdelkader, Zeinab Fathalla, Hossam Moharram, Taha F. S. Ali, Barbara Pierscionek

**Affiliations:** ^1^Department of Pharmaceutics, Faculty of Pharmacy, Minia University, Mina, Egypt; ^2^Department of Ophthalmology, Faculty of Medicine, Minia University, Minia, Egypt; ^3^Department of Medicinal Chemistry, Faculty of Pharmacy, Minia University, Mina, Egypt; ^4^School of Science and Technology, Nottingham Trent University, 50 Shakespeare Street, Nottingham NG1 4FQ, UK

## Abstract

With advances in refractive surgery and demand for cataract removal and lens replacement, the ocular use of nonsteroidal anti-inflammatory drugs (NSAIDs) has increased. One of the most commonly used NSAIDs is diclofenac (Diclo). In this study, cyclodextrins (CDs), *α*-, *β*-, *γ*-, and HP-*β*-CDs, were investigated with *in vitro* irritation and *in vivo* ulceration models in rabbits to reduce Diclo toxicity. Diclo-, *α*-, *β*-, *γ*-, and HP-*β*-CD inclusion complexes were prepared and characterized and Diclo-CD complexes were evaluated for corneal permeation, red blood cell (RBCs) haemolysis, corneal opacity/permeability, and toxicity. Guest- (Diclo-) host (CD) solid inclusion complexes were formed only with *β*-, *γ*-, and HP-*β*-CDs. Amphipathic properties for Diclo were recorded and this surfactant-like functionality might contribute to the unwanted effects of Diclo on the surface of the eye. Contact angle and spreading coefficients were used to assess Diclo-CDs in solution. Reduction of ocular toxicity 3-fold to16-fold and comparable corneal permeability to free Diclo were recorded only with Diclo-*γ*-CD and Diclo-HP-*β*-CD complexes. These two complexes showed faster healing rates without scar formation compared with exposure to the Diclo solution and to untreated groups. This study also highlighted that Diclo-*γ*-CD and Diclo-HP-*β*-CD demonstrated fast healing without scar formation.

## 1. Introduction

Cyclodextrins (CDs) are cyclic oligosaccharides with a hydrocarbon (water repellant) cavity and a hydrophilic outer surface composed of 6, 7, or 8 dextrose units forming the three parent cyclodextrins *α*-, *β*-, and *γ*-CDs. Depending on the number of dextrose units, the hydrophobic cavity varies in size and can accommodate various lipophilic moieties and form guest-host complexes. These complexes have been well-known as inclusion complexes and utilized in pharmacy and cosmetic industries to enhance water solubility and bioavailability of poorly soluble drugs and to improve palatability by reducing the bitterness of certain drugs [1, 2]. More recently, CDs have been employed to fabricate drug-loaded textiles for treatment of surface skin diseases due to psoriasis, fungal infections, and insect bites [3].

CDs have gained much popularity and are widely used in the pharmaceutical industry for systemic routes such as oral and parenteral dosage forms [4]. Orally administered CDs are considered to be nontoxic because of their lack of absorption from the gastrointestinal tract and *γ*- and hydroxypropyl- (HP-) *β*-CD having been used safely via the parenteral route [5]. It is well-known that faster onset of action and less gastrointestinal side effects are attributed to oral piroxicam-*β*-CD than piroxicam alone [4]. In addition, Dyloject® and Akis® are two relatively new commercially available injectable Diclo-HP-*β*-CD complexes that gave rapid and effective analgesia comparable to Voltaren® IM injection not containing CDs [4].

The use of CDs in ophthalmology has been recently reported for solubilization of insoluble drugs such as corticosteroids [6], medications for treating glaucoma [7] and immunosuppressive agents [8] and for enhancing ocular permeability of drugs through the extremely lipophilic corneal epithelial membrane [9]. For example, CDs have been more successfully used to solubilize dorzolamide at physiological pH and to offer comparable ocular bioavailability at low viscosity (3 to 5 centipoises), to that found with high viscosity (100 centipoises) eye drops as well as at low pH of 5.65 to solubilize 2% of dorzolamide [7]. However, the use of eye drops containing *α*-CD > 4% has been found to cause superficial epithelial toxicity and microerosion in rabbit corneal tissue [10]. These effects most likely result from the ability of CD, especially *α*- and *β*-CD, to extract cholesterol and other lipid components from cell membranes [11] leading to cellular disruption and enhanced drug permeation through the corneal epithelial membrane [9]. Conversely, *γ*- and hydroxypropyl-*β*-CD are better tolerated in ocular tissues and less likely to cause disruption of the corneal epithelial barrier [8, 9]. CDs have the potential to alter drug availability at the absorption site as well as to modify the rate of drug release and hence can be applied to reduce drug irritation caused by localized high concentrations. There are no reports of research that has critically assessed the ocular irritation potential from drug-CD complexes versus free drug solutions using *in vitro* irritation models.

With the advent of modern refractive and cataract surgeries, nonsteroidal anti-inflammatory drugs (NSAIDs) have been increasingly used in ophthalmology as a safer alternative to topical corticosteroids, avoiding serious side effects such as increasing IOP, cataractogenesis, risk of infection, and stromal melting [12]. NSAIDs can effectively reduce miosis, inflammation, pain, and scleritis and, more importantly, prevent and treat cystoid macular oedema associated with cataract surgeries [13, 14]. Diclofenac eye drops (0.1% *w*/*v*) and other NSAIDs such as ketorolac tromethamine (0.5% *w*/*v*), suprofen (1% *w*/*v*), flurbiprofen (0.03% *w*/*v*), and indomethacin (1%) are commercially available and widely used for multiple indications such as reducing pain and inflammation after ocular surgery and for seasonal allergic conjunctivitis [14]. Side effects and toxicities that have been widely reported with topical application of NSAIDs can range from transient burning, stinging, and conjunctival hyperaemia to more serious effects such as superficial keratitis, corneal erosion, corneal epithelial defect, and corneal ulceration and melting [12, 15, 16].

This study aimed to investigate a possible role of CDs for reducing local irritation and corneal toxicity of the most widely used NSAID, namely, diclofenac. Previous studies have sought to investigate corneal irritation potential from Voltaren Ophtha eye drops and cyclodextrins such as hydroxypropyl-*β*-CD using *in vitro* models such as hen's egg test-chorioallantoic membrane (HET-CAM), cytotoxicity, and haemolysis assay [17, 18]. Both *in vitro* and *in vivo* assessments for ocular irritation potential of Diclo with four different CDs, employing bovine corneal opacity and permeability (BCOP), RBC haemolysis and MTT assay using human primary corneal epithelial cells, and *in vivo* corneal healing in rabbits have been undertaken in this study. No reported investigations to date have considered the amphipathic properties of Diclo that are assessed in this work for surface tension and contact angle measurements using drop shape analysis.

## 2. Materials and Methods

### 2.1. Materials

Diclofenac sodium was donated by PSM Healthcare Pharma, Auckland, New Zealand. *α*-, *β*-, *γ*-, and HP-*β*-CD, cellophane membrane (MW-cut off 12,000-14,000 Dalton), nitro blue tetrazolium (NBT), and 3-(4,5-dimethylthiazol-2-yl)-2,5-diphenyltetrazolium bromide (MTT) were purchased from Sigma-Aldrich, UK. All other chemicals and reagents were of analytical grade and used as received.

### 2.2. Preparation of Diclo-CD Physical Mixtures

Equivalent amounts in mg of 1 : 1 molar ratios of Diclo and different types of CD (*α*-, *β*-, *γ*-, and HP-*β*-CD) were separately weighed and mixed uniformly in a porcelain dish with a spatula for 5 minutes. The physical mixtures were collected in glass vials and sealed and stored in a cool dry place for further use.

### 2.3. Preparation of Diclo-CD Complexes Using Solvent Evaporation Method

Diclo and different types of CD (*α*-, *β*-, *γ*-, and HP-*β*-CD) were weighed in 1 : 1 molar ratios and dissolved separately in methanol (20 ml) and deionized water (10 ml), respectively. The two solutions were mixed and magnetically stirred in 100 ml capacity-evaporating basins and allowed to completely evaporate at 60°C. The resulting solid complexes were left overnight in a desiccator for removal of residual moisture and pulverized and stored in glass vials for subsequent use.

### 2.4. Characterization of the Prepared Cyclodextrin Complexes

#### 2.4.1. Differential Scanning Calorimetry

Diclo-, *α*-, *β*-, *γ*-, and HP-*β*-CD and corresponding physical mixtures and complexes (amounts of 5 to 8 mg) were weighed separately in an aluminum pan; an aluminum lid was replaced and crimped using a pan press (Thermal Science, USA). The temperature of the pan was gradually raised from 25 to 300°C at a rate of 10°C/min using a differential scanning calorimeter (DSC) (Mettler Toledo DSC 822e0, Switzerland). Nitrogen gas was purged at a rate of 45 ml/min. Data were collected online using Mettler STARe software version 8.10, Switzerland.

#### 2.4.2. Fourier Transform Infrared Spectroscopy (FT-IR)

Amounts (2–4 mg) of Diclo-, *α*-, *β*-, *γ*-, and HP-*β*-CD and the physical mixtures and complexes were used to form a thin film covering a diamond window of the FT-IR spectrometer (Thermo Scientific Nicolet iS5, Thermo fisher, Madison, USA). The data were collected and analyzed using Omnic software (Omnic version 8.2, USA). The FT-IR spectra were registered at a spectral resolution of 2 cm^−1^ with an average of 20 scans and a scanning range of 4000 cm^−1^ to 600 cm^−1^.

#### 2.4.3. Molecular Docking

In order to predict the orientation within the cavity and/or rim and gain more insights into the stability/binding constants of Diclo with *α*-CD, *β*-CD, *γ*-CD, and HP-*β*-CD, molecular docking studies were performed using Molecular Operating Environment (MOE) 2014.09 software (Chemical Computing Group, Montreal, QC, Canada). The crystal structures of CDs were extracted from Protein Data Bank (PDB): *α*-CD (PDB code: 5E6Y), *β*-CD (PDB code: 5E6Z), and *γ*-CD (PDB code: 5E70) [19]. Since no crystal structure is available for HP-*β*-CD, the crystal structure of *β*-CD (PDB code: 5E6Z) was used as a template to build the 3D structure of HP-*β*-CD by substituting four primary hydroxyl groups with 2-hydroxypropyl radical, as described elsewhere [20]. The 3D crystal structure of Diclo was retrieved from crystallographic data available in the Cambridge structural database (Ref. Code: LIQFUN) [21]. The docking simulations were performed using the induced fit docking protocol. All other parameters were used with the default molecular operating environment (MOE) settings. The resulting docking poses were visually inspected, and the best energy pose for each type of the four Diclo-CD complexes was selected.

### 2.5. Preparation of Diclo Solution (0.1% *w*/*v*) and Its Equivalent from Diclo-CD Complexes

An amount of Diclo (10 mg) or equivalent to 10 mg from the prepared Diclo-CD complexes was dissolved in 10 ml of isotonic solution of phosphate buffer saline (PBS) pH 7.4 and sterile filtered through 0.22 *μ*m sterile syringe filters to prepare final solutions containing 0.1% *w*/*v* of Diclo. The prepared solutions were stored at 4°C until further use.

### 2.6. Evaluation of Diclo and Diclo-CD Solutions

#### 2.6.1. Contact Angle, Surface Tension, and Spreading Coefficient Measurements

The contact angle and surface tension for Diclo (0.1% *w*/*v*) and its equivalent from Diclo-CD solutions in PBS were performed according to our previously published work using a drop shape analyzer (goniometer) (Kruss Drop Shape Analysis, Hamburg, Germany) [22].

#### 2.6.2. Transcorneal Permeation Studies Using Excised Porcine Eyes

Excised porcine eyes were collected from a local abattoir (Jennings Butchery, Surbiton, UK). The cornea was dissected as previously described [22]. Franz diffusion cells were employed for ex vivo permeation, and the temperature was maintained at 35°C ± 0.5°C. The receptor chambers were filled (12 ml) with PBS, pH 7.4. The medium was constantly stirred using small magnetic bars. Volumes of 2 ml of each formulation were pipetted into the donor compartment providing a surface area of 1.7 cm^2^. Samples of 1 ml were withdrawn at predetermined time points for up to 8 h and replaced with the same volume of the medium without drug. The samples were analyzed at *λ*_max_ = 275 nm using a UV/visible spectrophotometer (Genway 7305, Hanwell, London, UK). Negative controls (in PBS without the drug) were placed on corneae and were withdrawn at the same time points as test samples.

The permeability coefficient (*P*_app,_ cm/s) was calculated using [23]:

(1) $$\begin{array}{c} P_{app}=\frac{\Delta Q}{\Delta t\left(3600\right){AC}_{o}}. \end{array}$$

∆*Q*/∆*t* is the permeability rate of Diclo across the excised porcine cornea; *C*_o_ is the initial Diclo concentration (*μ*g/cm^3^); *A* is the exposed surface area of the cornea (cm^2^). The value of 3600 represents the conversion of hours to seconds.

#### 2.6.3. Red Blood Cell (RBC) Haemolysis Assay

The RBC assay was based on DB-ALM protocol number 99 [24]. Fresh bovine blood samples were collected from ABP Guildford London, UK, and were mixed in a ratio of 1 in 10 with citrate buffer as anticoagulate. The citrated blood was further diluted to 4 : 10 volumes in PBS and then centrifuged at 1500 ×g, 4°C for 10 minutes. The supernatant was carefully discarded, and the pellets were washed with sterile PBS. A total of five washes were made. The final pellets were resuspended in PBS supplemented with 10 mmol/l glucose and stored in the fridge until further use. The test materials (Diclo-, *α*-, *β*-, *γ*-, and HP-*β*-CD and Diclo-, *α*-, *β*-, *γ*-, and HP-*β*-CD dispersed mixtures) were dissolved in PBS at the following final assay concentrations in mg/l (*w*/*v*): 1, 10, 100, 1000, and 10,000. One part of the RBC final suspension was added to 3 parts of the test material in PBS to give the aforementioned final assay concentrations. The mixtures were incubated for 60 minutes with agitation at room temperature. After incubation, the samples were centrifuged at 1500 ×g and 4°C for 1 minute and the extent of haemolysis was determined spectrophotometrically at 541 nm using a UV/visible spectrophotometer (Genway 7305, Hanwell, London, UK) and the percentage haemolysis estimated by comparison with a sample that was totally lysed with deionized water. The concentration of a test substance that induced the lysis of 50% of RBCs (*H*_50%_) was determined and used to evaluate the irritation potential of Diclo-CD mixtures.

#### 2.6.4. Bovine Corneal Opacity and Permeability (BCOP) Assay

Bovine eyes were obtained from a local slaughterhouse (ABP Guilford, London, UK). Eyes with corneal damage or abnormalities were discarded. Three different controls were used for validation purposes; sodium hydroxide (0.5 M) was used as a corrosive test substance, benzalkonium chloride (BKC) 1% *w*/*v* in PBS was used as a strong irritant control, and propylene glycol as a slight irritant. Diclo solution (0.1% *w*/*v*) and Diclo-CD solutions containing an amount equivalent to 0.1% *w*/*v* of Diclo were tested.

The extent of corneal damage was assessed by evaluating the opacity, followed by application of sodium fluorescein solution (2% *w*/*v* pH 7.4) to examine the integrity of the corneal epithelium, using an examination lamp and a cobalt blue filter (Leica, GmbH, Germany). Individual numerical scores for opacity, epithelial integrity (degree of staining), and epithelial detachment were reported elsewhere [25] and in more recently published work [26]. The sum score was estimated and the mean score for each of the three eyes was used to interpret the corneal irritation potential.

#### 2.6.5. Cytotoxicity Evaluation (MTT Assay)

Primary human corneal epithelial cells (ATCC pcs-700-010) from ATCC were seeded at approximately 2 × 10^4^ cells/well into 96 well plates (Nunc, Netherland) using corneal epithelial cell basal medium containing apotransferrin (5 mg/ml), epinephrine (1.0 mM) extract P (0.4%), hydrocortisone hemisuccinate (100 ng/ml), L-glutamine (6 mM), rh insulin (5 mg/ml), and CE growth factor (1 ml). The cells were allowed to establish for 48 hours prior to treatment. Media were removed and fresh media containing 5 different treatments were added. The treatments were Diclo (0.1% *w*/*v*) and Diclo-, *α*-, *β*-,*γ*-, and HP-*β*-CD solutions containing equivalent concentrations of Diclo 0.1% *w*/*v*. The medium served as the negative control and benzalkonium chloride (BKC) at a concentration of 0.01% *w*/*v* was used as the positive control. After 4 hours of treatment, the media were removed and the cells were washed twice with sterile PBS at 37°C and then further incubated with 200 *μ*l per well of 0.5 mg/ml 3-(4,5-dimethylthiazol-2-yl)-2,5-diphenyltetrazolium bromide (MTT) solution at 37°C. After incubation, the MTT solution was carefully removed and the wells were washed twice with sterile PBS. Finally, 200 *μ*l of dimethylsulfoxide (DMSO) was added to each well to lyse the cells. The cells were then gently agitated to mix the samples and analyzed on a TECAN Infinite M200 pro plate reader (Männedorf, Switzerland) at a wavelength of 540 nm. Experiments were performed in triplicate, and the average percentage cell viability was estimated.

#### 2.6.6. In Vivo Study (Alcohol Delamination and Corneal Epithelial Scrapping)

Specified amounts of Diclo or equivalent from Diclo-*γ*-CD- and HP-*β*-CD-dispersed mixtures were dissolved in Vigamox® eye drops to form Diclo 0.1% *w*/*v* solutions. Twenty-seven white albino rabbits, weighing between 2.0 and 2.5 kg, were used in the experiments. The rabbits were fed on balanced diet pellets and maintained on 12 h/12 h light/dark cycle in an air-conditioned room, at 28°C before the experiment. The experimental procedures were approved by Minia University Animal Ethics Committee (Minia, Egypt) and conformed to ethical guidelines.

The rabbits were divided into three groups with nine animals in each.  Table 1 summarizes the different types of treatment. Each treatment was initiated directly after induction of the corneal ulcer as a single drop instilled every 6 hours.

Prior to induction of ulcers, both eyes were locally anaesthetized with instillation of a single drop of Benox® eye drops (0.4% benoxinate hydrochloride) in each eye. A 6 mm ring was applied to the central corneal zone, and 20% *v*/*v* ethyl alcohol was applied inside the ring for 15 seconds to ease epithelial removal (delamination) followed by epithelial scrapping with a sterile scalpel blade. The ulcers were immediately stained with fluorescein, and the stain was visualized in a dark room using a hand-held indirect ophthalmoscope with a cobalt blue filter (Omega 500, Heine, Germany); the treatment was initiated as aforementioned.

#### 2.6.7. Statistical Analysis

Statistical analysis was performed with GraphPad Prism 6 (2014) software, using analysis of variance (ANOVA) with a Dunnett post hoc test for confidence intervals of 95% with statistical significance set at *p* < 0.05 in order to reveal statistical significant differences among contact angle, surface tension, spreading coefficient, transcorneal permeation parameters, cumulative BCOP scores, and % cell viability.

## 3. Results and Discussion

### 3.1. Differential Scanning Colorimetry

**Figure 1:**
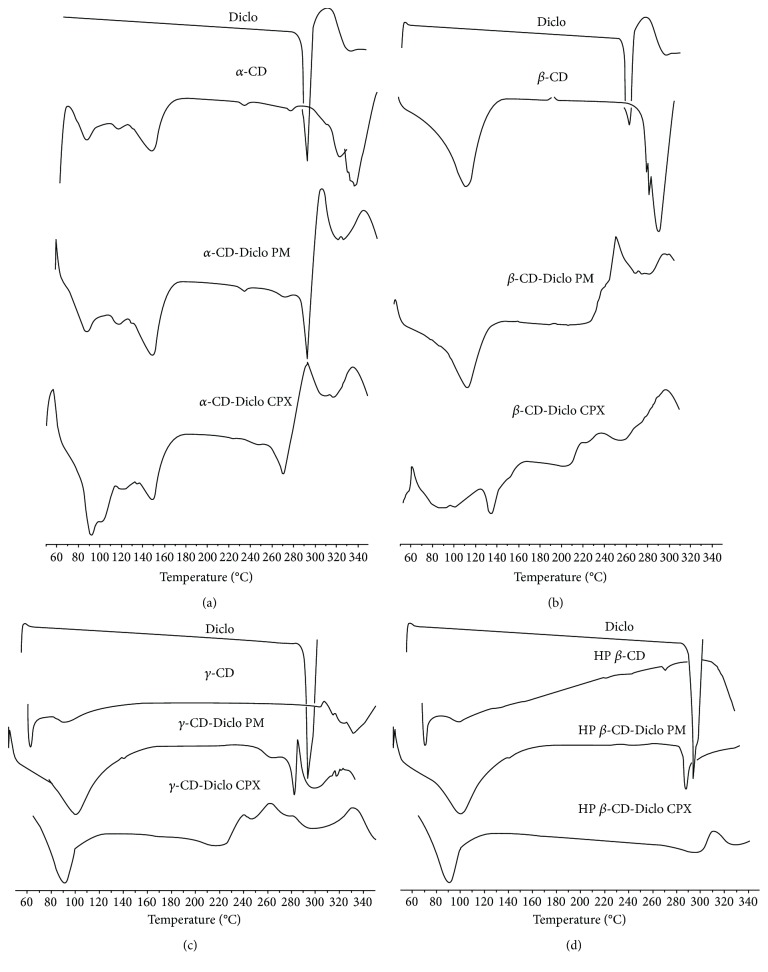
DSC curves for (a) Diclo, *α*-CD, *α*-CD-Diclo (PM), and dispersed (CPX); (b) Diclo, *β*-CD, *β*-CD-Diclo (PM), and dispersed (CPX); (c) Diclo, *γ*-CD, γ-CD-Diclo (PM), and dispersed (CPX); and (d) Diclo, HP-*β*-CD, and HP-*β*-CD physical (PM) and dispersed (CPX) mixtures.

DSC was used to study the crystallinity of Diclo and the possibility of formation of Diclo-CD inclusion complexes. Figures 1(a)‑1(d) shows the thermal behavior of Diclo with different types of CDs (*α*-, *β*-, *γ*-, and HP-*β*-CD), respectively. Diclo demonstrates a strong thermal endothermic event at 290°C caused by melting of diclofenac sodium. This strong melting peak was reduced in intensity with *α*-CD PM and complexes, and it completely disappeared with *β*-, *γ*-, and HP-*β*-CDs. The complete disappearance of the drug peak with CDs strongly suggests molecular dispersion and formation of molecular inclusion complexes of Diclo with *β*-, *γ*-, and HP-*β*-CDs. Shifting of the melting peak to the lower end of the temperature scale and formation of a lower intensity melting peak may indicate the formation of partial/incomplete inclusion complexes of Diclo with *α*-CD and could be caused by poorer fitting of the relatively bulky diclofenac within the smallest cavity size of 6-sugar units *α*-CD, compared with the higher 7- and 8-sugar units *β*- and HP-*β*-CDs, and *γ*-CD, respectively. The DSC curves of the physical mixtures and the dispersed mixtures that were prepared by the solvent evaporation technique did not show any marked differences because the heat supply during the DSC procedure can provide sufficient power equivalent to that of the solvent evaporation, which brings drug molecules and CD into intimate molecular dispersion/complexation. Similar behavior with other water-soluble carriers has been reported [27].

### 3.2. FT-IR Spectroscopy

**Figure 2:**
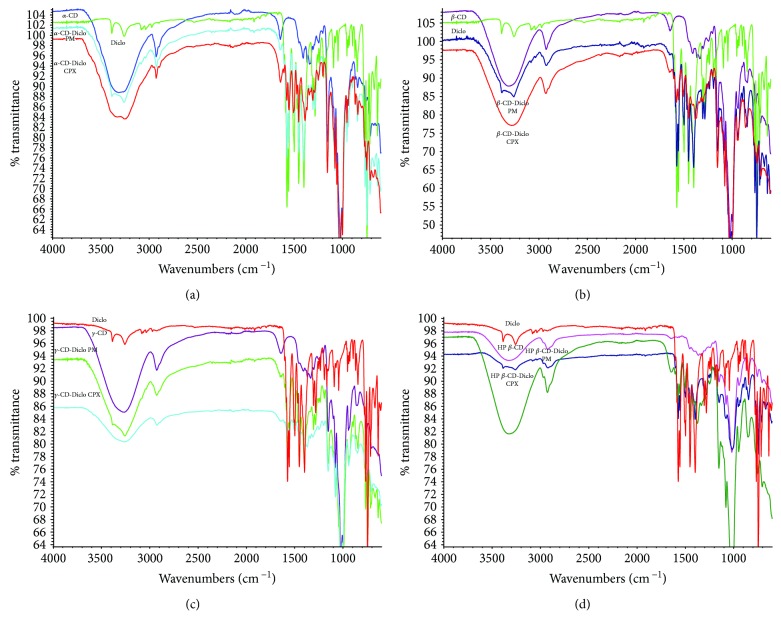
FT-IR spectra for (a) Diclo, *α*-CD, *α*-CD-Diclo (PM), and dispersed (CPX); (b) Diclo, *β*-CD, *β*-CD-Diclo (PM), and dispersed (CPX); (c) Diclo, *γ*-CD, *γ*-CD-Diclo (PM), and dispersed (CPX); and (d) Diclo, HP-*β*-CD, and HP-*β*-CD physical (PM), and dispersed (CPX) mixtures.

FT-IR spectroscopy was utilized to study any potential interactions between Diclo and different CDs, as shown in Figures 2(a)‑2(d). The IR spectrum of Diclo showed a strong characteristic peak at 1600 cm^−1^ assigned for *C* =*O*, two IR absorption peaks resulting from secondary amine N-H, and aromatic stretching *C* = *C*-bands at 3400 and 3300 cm^−1^ [28]. These spectral regions are of interest for investigating the possibility of formation of inclusion complexes [29]. All CDs that showed a characteristic broad stretching absorption peak appearing at 3550 to 3200 cm^−1^ were assigned to different alcoholic O-H of the cyclic sugar units of *α*-, *β*-, *γ*-, and HP-*β*-CDs (Figures 2(a)-2(d)). The physical mixtures (PM) were a superimposition of the two spectra of Diclo and corresponding CDs when their characteristic peaks were unchanged; this occurred with Diclo-*α*, *β*-, *γ*-, and HP-*β*-CD PM. Apart from Diclo-*α*-CD, significant changes in the IR region of 3550–3200 cm^−1^ were seen with Diclo-, *β*-, *γ*-, and HP-*β*-CD-dispersed mixtures prepared by the solvent evaporation method. Additionally, the strong absorption bands of *C* = *O* group of Diclo were markedly reduced in intensity and showed a frequency shift with all CDs. These changes can be explained by characteristic guest-host interactions and formation of inclusion complexes of Diclo with *β*-, *γ*-, and HP-*β*-CD-dispersed mixtures. Conversely, Diclo- and *α*-CD complex still shows a small peak at 3300, which might be indicating the free drug, as well as incomplete complexation for *α*-CD. These results concurred with those obtained using DSC.

### 3.3. Molecular Docking

**Figure 3:**
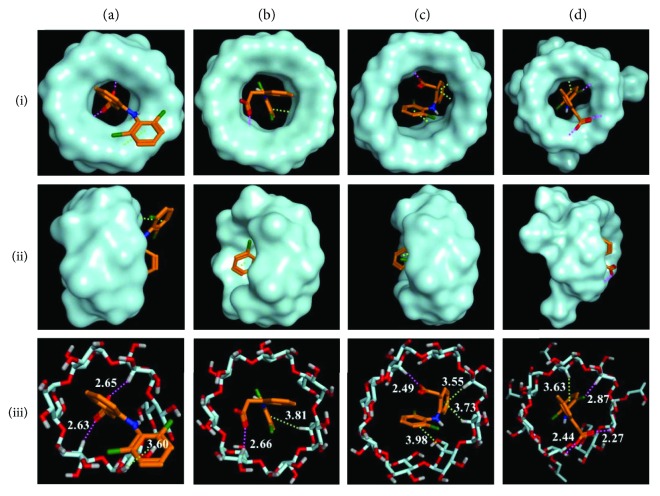
The predicted orientations and binding interactions of diclofenac within the cavity of four cyclodextrins (a) *α*-CD, (b) *β*-CD, (c) *γ*-CD, and (d) HP-*β*-CD from the top view of the wide edge (i), side view (ii), and as stick molecular depiction (iii). Hydrogen bonds and hydrophobic interactions are demonstrated as magenta and green dashed lines, respectively.

To gain more insights into the binding mode and binding constants between Diclo and individual CDs, molecular docking between Diclo and *α*-, *β*-, *γ*-, and HP-*β*-CDs was performed. Molecular docking simulation is the best method to predict drug (guest) orientations, molecular fitting and interactions into/onto the host (CDs) hydrophobic cavity, and hydrophilic rims at the molecular level [30].  Figure 3 summarizes the molecular docking results. The binding constants estimated for Diclo-, *α*-, *β*-, *γ*-, and HP-*β*-CDs complexes were −4.3 kcal/mol, −4.4 kcal/mol, −4.8 kcal/mol, and −5.2 kcal/mol, and the force of binding of Diclo with CDs was in the following order: HP-*β*-CD > > *γ*-CD > *β*-CD > *α*-CD.

Electrostatic interactions (H-bonding) between carboxylate group of Diclo and the hydrophilic rim (hydroxyl groups: OH) of *α*-CD Figure 3, ia and ic) were recorded. However, the size of the *α*-CD cavity was too small to host the dichlorophenyl ring of Diclo (the dichlorophenyl ring was completely outside the CD cavity) (Figures 1(a) and 2(a)). This led to a less stable complex with a binding constant of −4.3 kcal/mol. These results correlated well with DSC and FT-IR studies (Figures 1(a) and 2(a)) where the Diclo melting peak appeared with the processed Diclo- and *α*-CD mixture indicating a negligible physicochemical interaction. Conversely, both *γ*- and HP-*β*-CDs had suitable cavity sizes for hosting Diclo in their hydrophobic cavity (Figure 3, ic, iic, iiic, id, iid, and iiid ) where the dichlorophenyl ring was completely buried in *γ*- and HP-*β*-CDs. The cavity size of HP-*β*-CDs is larger than that of *α*-CD, and it contains an additional extension arising from the hydroxypropyl substitution when compared with *β*-CD. The two hydrogen bonds as well as the halogen-hydrogen bond could also contribute to a very stable complex with binding energy of −5.2 kcal/mol. The cavity size of *γ*-CD is sufficiently large to host Diclo with three hydrophobic interaction sites that lead to a stable complex with binding energy of −4.8 kcal/mol (Figure 3, iiic).

While the *β*-CD cavity size is larger than that of *α*-CD, the dichlorophenyl ring could not be entirely hosted within it (Figure 3, iiib). Instead, the two aromatic rings of Diclo were bent with a torsional angle of 69 degrees [31]; the dichlorophenyl was partly outside the pocket/cavity as shown in Figure 3, ib and Figure 3, iiib. This produces a fairly stable complex with binding energy of −4.4 kcal/mol. Similar results of the crystal structures of Diclo with *β*-CD have been reported elsewhere [32].

### 3.4. Contact Angle, Surface Tension, and Spreading Coefficient

Table 2 shows the surface tension for Diclo solution (0.1% *w*/*v* in PBS) and equivalent amounts of Diclo-, *α*-, *β*-, *γ*-, and HP-*β*-CD-dispersed mixtures that constitute the Diclo solution of 0.1% *w*/*v*. The *γ* value recorded for Diclo solution (0.1% *w*/*v*) was 52 mN/m. This is a significant decrease in the surface tension of the Diclo solution compared with the solvent (PBS) which was 76 mN/m and suggests that Diclo in solution has a surfactant-like property. The dichloride substituted aromatic ring with the NH linker to benzoate structure can explain the amphipathic nature and surface active properties of diclofenac sodium. Amphipathic properties have been reported with structurally similar drugs such as chlorpromazine, diphenhydramine, chlordiazepoxide, and chlorcyclizine [33].

While the exact mechanism of the onset of corneal melting remains unknown [12], the above-reported surface active properties attributed to Diclo may provide some insight into how the topical ocular administration of Diclo produces irritant/toxic effects. It could be caused by exposure of the ocular surface to relatively high local concentrations of a drug with surface active properties that can induce emulsification and/or a sloughing of the extremely lipophilic corneal epithelium.

The surface tension of Diclo-, *α*-, *β*-, *γ*-, and HP-*β*-CD solutions exhibited a significant (*p* < 0.05) increase in the surface tension values (60–65.5 mN/m). Furthermore, Diclo-CD solutions showed significant increases in contact angle (*θ*) and associated significant decreases in spreading coefficients (*S*). This supports the formation of complexes of Diclo with *α*-, *β*-, *γ*-, and HP-*β*-CDs in solution that may result in reducing the surface active properties of the drug because the hydrophobic part of Diclo is found within the cavity of CDs. These results are consistent with the docking calculations: various H-bonding and hydrophobic interactions of carboxylate group and/or dichlorophenyl ring of Diclo with the hydrophilic rims and hydrophobic cavities of CDs were recorded (Figure 3, ia, iia, iiia, ib, iib, iiib, ic, iic, iiic, id, iid, and iiid ). Furthermore, the behavior of complexes of Diclo with CDs in solutions was reported to be different than their behavior in the solid state (1 : 1 molar complexation) [34, 35]. The two aromatic rings have been reported to be involved 1 : 2 CD complexation. This could explain why the contact angle and surface tension measurements for Diclo-, *α*-, *β*-, *γ*-, and HP-*β*-CD solutions did not show statistically significant differences (*p* > 0.05).

### 3.5. Transcorneal Permeation Study

**Figure 4:**
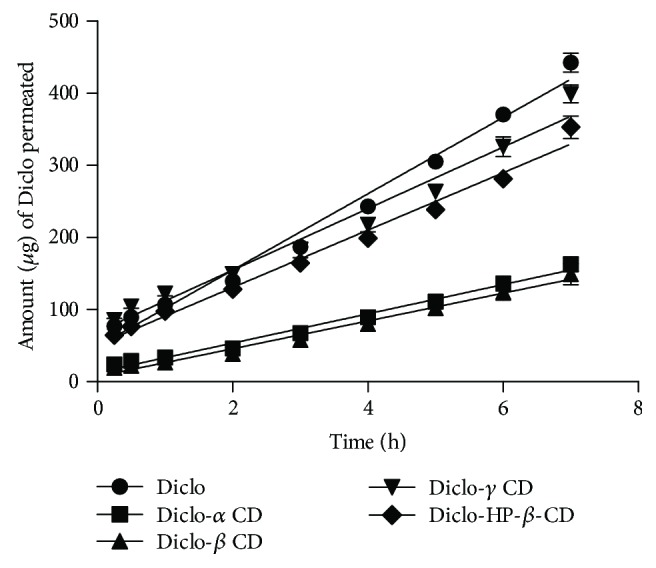
Transcorneal permeation profiles of Diclo form drug solution and Diclo-CD solutions. Results presented as mean values ± SD, *n* = 3.

Porcine corneal permeation profiles of Diclo from free drug solutions and different Diclo-, α-, *β*-, *γ*-, and HP-*β*-CD complexes are outlined in Figure 4. Permeation parameters (steady-state flux and apparent permeability coefficient (*P*_app_)) of the different Diclo solutions tested are given in Table 2. Both steady-state flux and *P*_app_ values (19–41.5 *μ*g/h and 4–9 cm/s) for Diclo permeated from different Diclo-, *α*-, *β*-, *γ*-, and HP-*β*-CD solutions showed significant controlled/sustained permeation of Diclo from *α*-, *β*-, *γ*-, and HP-*β*-CD solutions compared with those values estimated for free Diclo solution (53 *μ*g/h and 12 cm/s). Cyclodextrins are extremely hydrophilic and cannot permeate through lipophilic corneal barriers [1, 10] suggesting that Diclo molecules had to be liberated from the guest-host complex in order to permeate through the corneal barrier and that the *P*_app_ of Diclo is dependent on the binding forces of the guest-host complexes [18].

Diclo complex with *β*-cyclodextrin has been reported to transfer with higher rate through the cornea compared to free drug in the previous studies [36]. Valls et al. used a different device to study transcorneal permeation through rabbit's cornea. While the setup was developed in-house and to our knowledge this device is not available commercially, this setup can take into consideration tear dynamics (washings) and other ocular pharmacokinetic parameters. It is worth noting that Franz diffusion cells are a static *in vitro* permeation model that cannot perfectly mimic *in vivo* tear/ocular dynamics that represent a major factor for drug loss of topically administered eye drops on the surface of the eye. Other positive features resulting in prolonging precorneal residence and mucoadhesion cannot be taken into account while using this model. Therefore, faster permeation rates from drug solutions have consistently been reported with this model compared with many other formulations [22, 37].

More pertinently, the results showed that not all CDs used provided equally controlled Diclo permeation through excised porcine corneae. While Diclo-, *α*-, and *β*-CDs showed the lowest permeation rate (*P*_app_) with 2.4-fold and 2.8-fold decreases compared with free Diclo solution, Diclo-, *γ*-, and HP-*β*-CD solutions showed markedly higher (*P*_app_) compared with the previous two CD congers. Both Diclo-, *γ*-, and HP-*β*-CD recorded (*P*_app_) 1.27-fold lower than free Diclo solution. Paradoxically, despite the docking calculations, showing that Diclo-, *γ*-, and HP-*β*-CDs were the most stable complexes; the drug transcorneal permeation from Diclo-, *γ*-, and HP-*β*-CD complexes was slightly faster than that from Diclo-, *α*-, and *β*-CD complexes. It may be that with ex vivo permeation, the binding forces are not the only factors that affect permeation rates through the cornea.

### 3.6. RBC Haemolysis Assay

**Figure 5:**
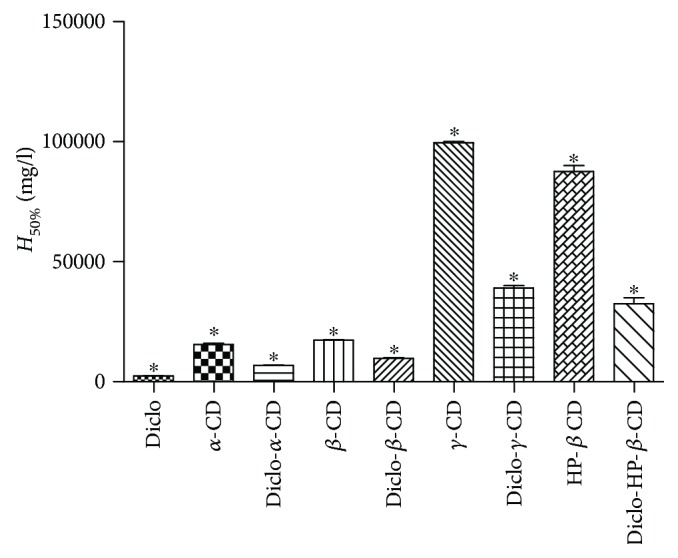
RBC haemolysis potential expressed in *H*_50%_ for Diclo solution and Diclo- *α*-, *β*-, *γ*-, HP-*β*-CD solutions. Results are expressed as mean values ± SD, *n* = 3. ∗ denotes statistically significant differences (*p* < 0.05).

RBC assay is an *in vitro* test that has been used widely for testing ocular irritation potential of ophthalmic pharmaceutical and cosmetic ingredients and surfactants [38, 39]. Acute cytotoxicity due to cell lysis, corneal erosion, and deepithelisation is well correlated with haemolytic activity and RBC lysis of test substances. The RBC assay has been reported to be correlated with the *in vivo* rabbit Draize test [10, 39].  Figure 5 shows the concentrations (*H*_50_) of the test substance that showed an absorbance value equivalent to 50% haemolysis of RBCs. The estimated *H*_50%_ for Diclo was 2500 mg/l, which is equal to 2.5 times of the drug concentration in commercial eye drops (0.1% *w*/*v*). The *γ*- and HP-*β*-CDs recorded *H*_50%_ at extremely high concentrations and can be considered as essentially nonirritant. Nevertheless, the *α*- and *β*-CDs were deemed to cause haemolysis at lower concentrations compared with *γ*- and HP-*β*-CDs. These findings support previous results indicating that *α*- and *β*-CDs can extract cholesterol and other lipid components of cell membranes thereby contributing to cell lysis [10, 11, 40]. It is worth noting that inclusion of Diclo into the cavities of the CDs by formation of guest-host complexes may have masked the inherent RBC haemolysis potential of Diclo. For example, *H*_50%_ recorded for Diclo-, *α*-, *β*-, *γ*-, and HP-*β*-CDs ranged from 7000 to 40,000 mg/l compared with a much lower *H*_50%_ value (2500 mg/l) for Diclo alone. This was accompanied by a reduction of cell lysis and possible corneal erosion by 3 to 16 times, compared with using the free drug alone.

### 3.7. BCOP Assay

**Figure 6:**
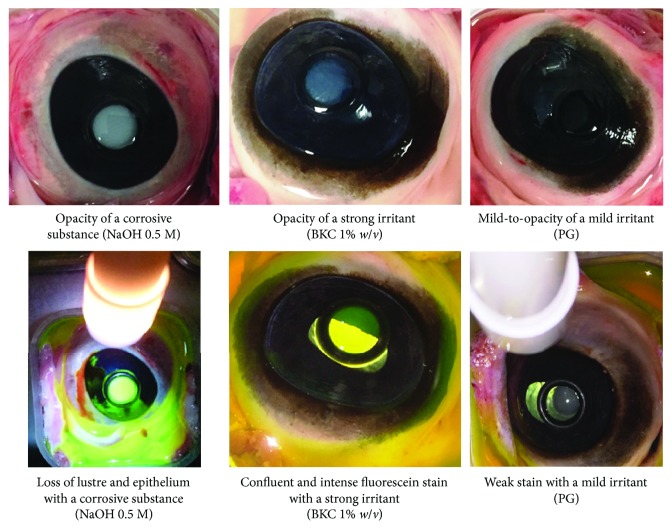
Degree of corneal opacity (upper) and fluorescein permeability (lower) used to score the test substances (corrosive (sodium hydroxide 0.5 M), strong irritant (benzalkonium chloride 1%), and mild irritant (propylene glycol) models).

The use of BCOP assay has been validated and approved by the Scientific Advisory Committee of the European Centre for the Validation of Alternatives (ECVAM). The BCOP assay is widely used across the cosmetic and pharmaceutical industries to test the ocular irritation potential of surfactants, pharmaceutical ingredients, and finished products [41–43]. The BCOP assay uses the assessment of corneal opacity and fluorescence intensity as an indication of degree of the disruption of the corneal barrier after exposure to the test material (Figure 6).

**Figure 7:**
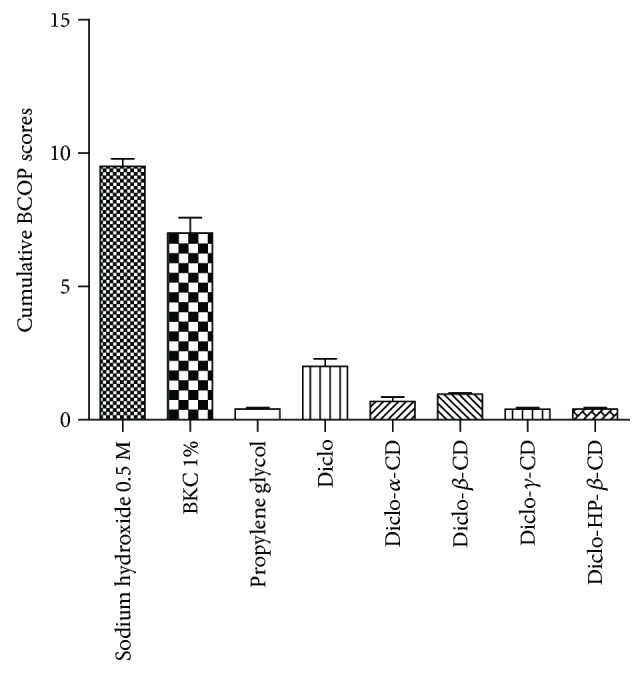
Cumulative BCOP scores for the three controls, Diclo, and different Diclo-CDs. Results presented as mean values ± SD, *n* = 3.

Figure 7 shows the cumulative BCOP scores of corneal opacity and epithelial integrity recorded for corrosive, strong, and mild irritant control and test substance Diclo solution and Diclo-, *α*-, *β*-, *γ*-, HP-*β*-CD solutions. The cumulative score for Diclo solution recorded 1.5 corresponding to mild-to-moderate irritants whereas Diclo-, *α*-, *β*-, *γ*-, HP-*β*-CD solutions recorded scores 0.5 to 1 corresponding to none-to-mild irritants with Diclo-, *γ*-, and HP-*β*-CD solutions exhibited the lowest cumulative scores. These results accord with the results from the RBC haemolysis assays.

### 3.8. Cytotoxicity Evaluation (MTT Assay)

**Figure 8:**
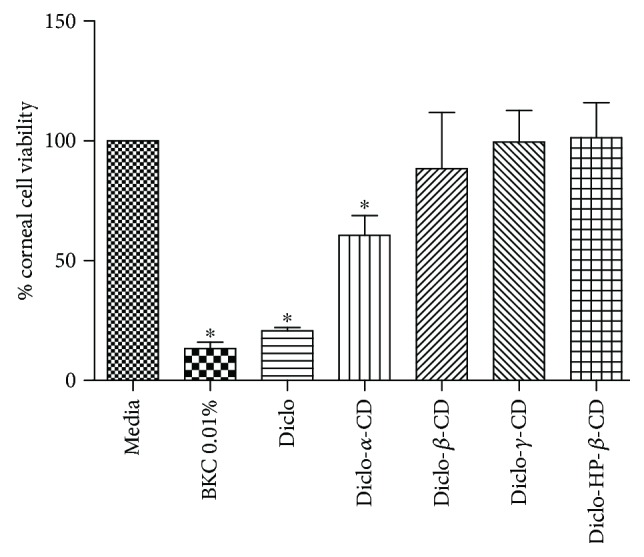
Percentage (%) cell viability for human primary corneal epithelial cells exposed to the test substances for 3 hours. Results are expressed as mean values ± SD, *n* = 3. ∗ denotes statistically significant differences (*p* < 0.05).

Percentage (%) corneal epithelial cell viability after a 4-hour exposure to various treatments is shown in Figure 8. BKC was used as a positive control and showed extremely low cell viability (13%) and was deemed to be cytotoxic at the duration tested in this study [44–46]. Diclo recorded corneal cell viability of 21% indicating poor cell viability and these results concur with the other two *in vitro* ocular toxicity models (BCOP and RBC haemolysis assays) and support previous reports on the harmful effects of topical application of Diclo to the corneal epithelium [13, 14]. The % cell viability estimated for Diclo-, *α*-, *β*-, *γ*-, and HP-*β*-CDs that contained an equivalent concentration of Diclo 0.1% *w*/*v* was significantly increased from 3-fold to 5-fold (*p* < 0.01) compared with free Diclo alone. While there were slight decreases in cell viability after exposure to Diclo-, *α*-, and *β*-CDs, these were correlated with the previous results that showed *α*- and *β*-CDs are less tolerated by the ocular surface, compared with *γ*- and HP-*β*-CDs. The latter can be considered as practically nonirritant and were able to mask the acute ocular toxicity of free Diclo solution.

### 3.9. *In Vivo* Study

**Figure 9:**
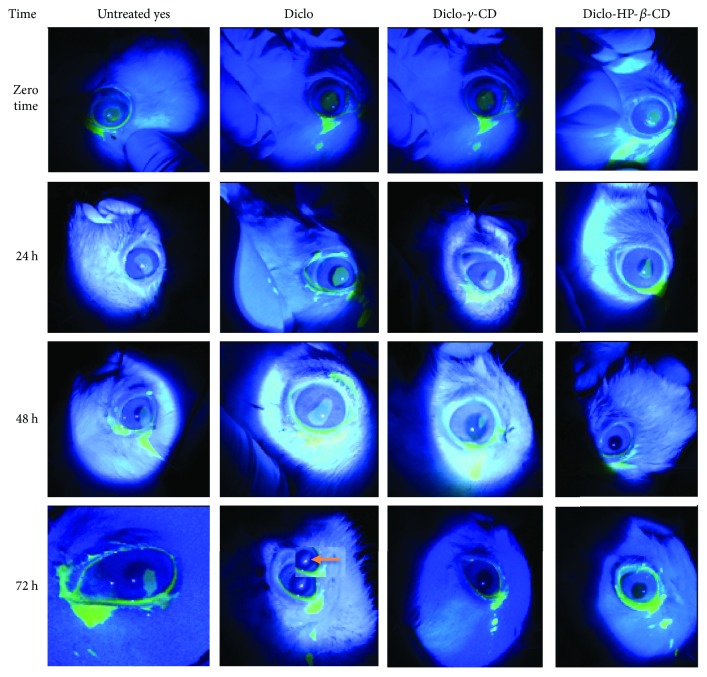
Progressive changes in alcohol-induced corneal ulcers in rabbit eyes where left eyes were untreated and right eyes were treated with Diclo, Diclo-*γ*-CD, and Diclo-HP-*β*-CD just after induction of ulceration. Of note is the corneal opacity (nebula) in the Diclo eye after 72 hours (as marked with an arrow).

Induction of corneal epithelial debridement by alcohol-assisted removal of corneal epithelium was adopted in this study as clinically relevant to the type of corneal wounds created with photorefractive keratectomy [47, 48].  Figure 9 shows a range of fluorescein stained corneal ulcers and corneal healing over time. Both eyes in group I showed complete healing in four days but with a nebula/scar in the right eye that received Vigamox eye drops with the anti-inflammatory drug Diclo 0.1% *w*/*v* (the nebula, seen at 72 hours, is indicated by an arrow). Group II and group III showed markedly faster healing rates; five rabbits out of 9 demonstrated complete corneal healing in 2 days without scar formation. These results can be ascribed to the following possibilities:

- Diclo-CDs may have lower direct irritation potential and lower toxicity by masking the inherent surfactant-like characteristics of Diclo.
- Subjecting the corneal ulcer to a transient high local concentration of free Diclo solution was avoided when Diclo was instilled as inclusion complexes with *γ*- and HP-*β*-CDs.
- Diclo-CDs may prolong precorneal residence time and enhance ocular bioavailability, compared with instillation of free Diclo eye drops. The literature indicates that dorzolamide-CD eyes drops with low viscosity (3 to 5 centipoises) exhibited comparable bioavailability with commercially viscous (100 centipoises) dorzolamide eye drops (Trusopt®) [7].

## 4. Conclusions

Ocular toxicity due to Diclo has been well reported and attributed solely to pharmacological factors such as inhibition of cyclooxygenase and/or upregulation of metalloproteinase matrix [17]. In this study, we report for the first time the possible toxicity of Diclo due to surfactant-like functionality and a formulation approach to significantly reduce/mask these undesirable characteristics using CD inclusion complexation. Diclo and *β*-, *γ*-, and HP-*β*-CDs can form guest-host inclusion complexes with different capacities for permeating through porcine corneae. The *α*- and *β*-CDs were deemed to be less effective at reducing the ocular unwanted toxicities of Diclo resulting from inherent ability to extract cholesterol and lipid components from the lipophilic corneal cell membranes. Contact angles, surface tensions, and spreading coefficients, measured for the first time, confirmed guest-host complex formation in solutions for the amphipathic drug Diclo. *In vitro* toxicity model RBC haemolysis and BCOP assays indicated the irritation potential from CD formulations, and the results were well correlated with those from the MTT cytotoxicity assay. The *γ*- and HP-*β*-CDs offer potential as carriers for effectively diminishing Diclo ocular toxicities. These two CD complexes exhibited a marked reduction in RBC haemolysis and significant increase in cell viability compared with Diclo solution alone. Diclo-*γ*- and Diclo-HP-*β*-CDs greatly enhance corneal wound closure without scar formation, compared with delayed corneal wound repair and scar formation with Diclo solution alone.

## Figures and Tables

**Table 1:** Summary of the rabbit groups recruited in the study and their treatments.

Group	Left eye	Right eye
Group 1	Vigamox eye drops	Vigamox eye drops containing 0.1% *w*/*v* Diclo
Group 2		Vigamox eye drops containing Diclo-*γ*-CD equivalent to 0.1% *w*/*v* Diclo
Group 3		Vigamox® eye drops containing Diclo-HP-*β*-CD equivalent to 0.1% *w*/*v* Diclo

**Table 2:** Contact angle, surface tension measurements, spreading, flux, and apparent permeability coefficient of diclofenac sodium from solution and cyclodextrin complexes. Results presented as mean values ± SD, *n* = 3. ∗ denotes statistically significant differences (*p* < 0.05).

Formulation	Contact angle (*θ*)	Surface tension (mN/m)	Spreading coefficient	Steady-state flux (*μ*g/h)	*P*_app_ × 10^−6^ (cm/s)
Diclo	34 ± 0.65^∗^	52.0 ± 2.5^∗^	−8.9 ± 2.00^∗^	53 ± 1.5^∗^	12.0 ± 0.5^∗^
Diclo-*α*-CD	52 ± 0.55	65.5 ± 1.0	−25.2 ± 1.0	21 ± 0.7	5.0 ± 0.5
Diclo-*β*-CD	50 ± 0.45	61.5 ± 2.0	−22.0 ± 1.5	19 ± 2.8	4.3 ± 1.0
Diclo-*γ*-CD	52 ± 0.80	60.35 ± 2.8	−23.2 ± 1.5	41.5 ± 3.5	9.4 ± 2.0
Diclo-HP-*β*-CD	53 ± 0.55	61.33 ± 2.0	−24.4 ± 2.0	41 ± 4.5	9.3 ± 2.0
